# The importance of climatic factors and outliers in predicting regional monthly campylobacteriosis risk in Georgia, USA

**DOI:** 10.1007/s00484-014-0788-6

**Published:** 2014-01-24

**Authors:** J. Weisent, W. Seaver, A. Odoi, B. Rohrbach

**Affiliations:** 1 Department of Comparative Medicine, The University of Tennessee, Knoxville, TN USA; 2 Department of Statistics, Operations and Management Science, The University of Tennessee, Knoxville, TN USA; 3 Department of Biomedical and Diagnostic Sciences, College of Veterinary Medicine, The University of Tennessee, 205A River Drive, Knoxville, TN 37996 USA

**Keywords:** Time series, Forecasting, Climatic factors, Campylobacteriosis risk, Control charting

## Abstract

Incidence of *Campylobacter* infection exhibits a strong seasonal component and regional variations in temperate climate zones. Forecasting the risk of infection regionally may provide clues to identify sources of transmission affected by temperature and precipitation. The objectives of this study were to (1) assess temporal patterns and differences in campylobacteriosis risk among nine climatic divisions of Georgia, USA, (2) compare univariate forecasting models that analyze campylobacteriosis risk over time with those that incorporate temperature and/or precipitation, and (3) investigate alternatives to supposedly random walk series and non-random occurrences that could be outliers. Temporal patterns of campylobacteriosis risk in Georgia were visually and statistically assessed. Univariate and multivariable forecasting models were used to predict the risk of campylobacteriosis and the coefficient of determination (*R* ^2^) was used for evaluating training (1999–2007) and holdout (2008) samples. Statistical control charting and rolling holdout periods were investigated to better understand the effect of outliers and improve forecasts. State and division level campylobacteriosis risk exhibited seasonal patterns with peaks occurring between June and August, and there were significant associations between campylobacteriosis risk, precipitation, and temperature. State and combined division forecasts were better than divisions alone, and models that included climate variables were comparable to univariate models. While rolling holdout techniques did not improve predictive ability, control charting identified high-risk time periods that require further investigation. These findings are important in (1) determining how climatic factors affect environmental sources and reservoirs of *Campylobacter* spp. and (2) identifying regional spikes in the risk of human *Campylobacter* infection and their underlying causes.

## Introduction

*Campylobacter* has been identified as a leading cause of human gastroenteritis in developed nations (Allos and Taylor 1998; Altekruse and Swerdlow 2002) with an estimated 1 % of the US population infected annually (Tauxe 2001). The bacteria are found throughout the natural environment, as well as in the gastrointestinal tracts of animals and birds (Snelling et al. 2005; Humphrey et al. 2007). Due to geographic diversity in land use, drinking and recreational water sources, and human behavior, establishing associations between campylobacteriosis risk and definitive environmental sources is difficult (Jepsen et al. 2009; Hartnack et al. 2009; Hearnden et al. 2003; Kovats et al. 2005; Miller et al. 2004; Bi et al. 2008).

The incidence of campylobacteriosis varies seasonally and geographically in temperate regions, and tends to be highest during summer months (Allos and Taylor 1998; Nylen et al. 2002; Tam et al. 2006). Variation in disease risk may be due to the effects of temperature and rainfall on the survival and reproduction of *Campylobacter* in the environment or on foods sources (Patrick et al. 2004; Fleury et al. 2006; Bi et al. 2009). Disease forecasting may provide clues as to how direct and indirect sources of transmission are affected by climate variation in different geographic regions (Bi et al. 2008). Specifically, investigating associations between temperature and precipitation patterns with the incidence of human campylobacteriosis may help focus efforts to identify the sources of infection and transmission, improve the accuracy of local forecasting, and provide an early warning system to alert epidemiologists and public health officials of potential epidemics (Nobre et al. 2001; De Greeff et al. 2009; Altizer et al. 2006; Hartnack et al. 2009). Research has shown that the seasonal patterns in campylobacteriosis risk vary among geographically diverse regions, yet are consistent within regions over time (Weisent et al. 2010; Kovats et al. 2005; Lal et al. 2012). Investigators have modeled the effect of climate on campylobacteriosis in Europe, Canada, Australia, and New Zealand (Bi et al. 2008; Kovats et al. 2005; Fleury et al. 2006; Hearnden et al. 2003; Allard et al. 2010). However, divisional models that incorporate temperature and precipitation have not been investigated in the United States.

Infectious disease patterns may change or behave erratically in response to biological and anthropogenic factors. Such changes can result in outliers—non-random deviations from the typical time series pattern. Outlier data points can take the form of a pulse, step or ramp, decay, or signify a more complicated directional change in the series. In surveillance data, change points may indicate outbreaks, geographic variation in reporting, and policy or prevention procedural changes. Outliers can greatly affect forecasting accuracy, and are particularly problematic for prediction when they occur near the end of a series or in the holdout (Bergmeir and Benitez 2012). Control charting can be used to detect outliers that can then be incorporated into models to understand and improve process performance as well as improve specification in forecasting and epidemic alert systems (Alwan 1988; Benneyan 2001). In this case study, some suggestions are made to provide practical ways of dealing with outlier issues in surveillance data.

The objectives of the research were to (1) assess temporal patterns and variations in campylobacteriosis risk in Georgia’s nine climatic divisions, (2) compare univariate time series forecasting models with those that incorporate exogenous climate variables (precipitation and temperature), and (3) investigate alternatives to supposedly random walk series and non random occurrences that could be outliers. We hypothesize that regional variations in temperature and precipitation affect campylobacteriosis risk over time. Study findings provide early indications of irregularities in disease incidence and therefore guide disease control programs that are based on empirical forecast results (Nobre et al. 2001; Williamson and Weatherby Hudson 1999; Myers et al. 2000; Benschop et al. 2008).

## Methods

### Study area

The study was conducted in the state of Georgia which covers almost 87,000 km^2^ and is characterized by a humid subtropical climate that varies greatly with topography (sea level to 1,433 m in elevation). Approximately 114 cm of rain fall in the central regions and 180 cm fall annually in the northeast mountains (Netstate 2012). Summers are hot and humid with an average daily temperature close to 32 °C and winters tend to be mild, but are cooler in the mountains and piedmont than in the coastal and southernmost regions of the state.

**Fig. 1:**
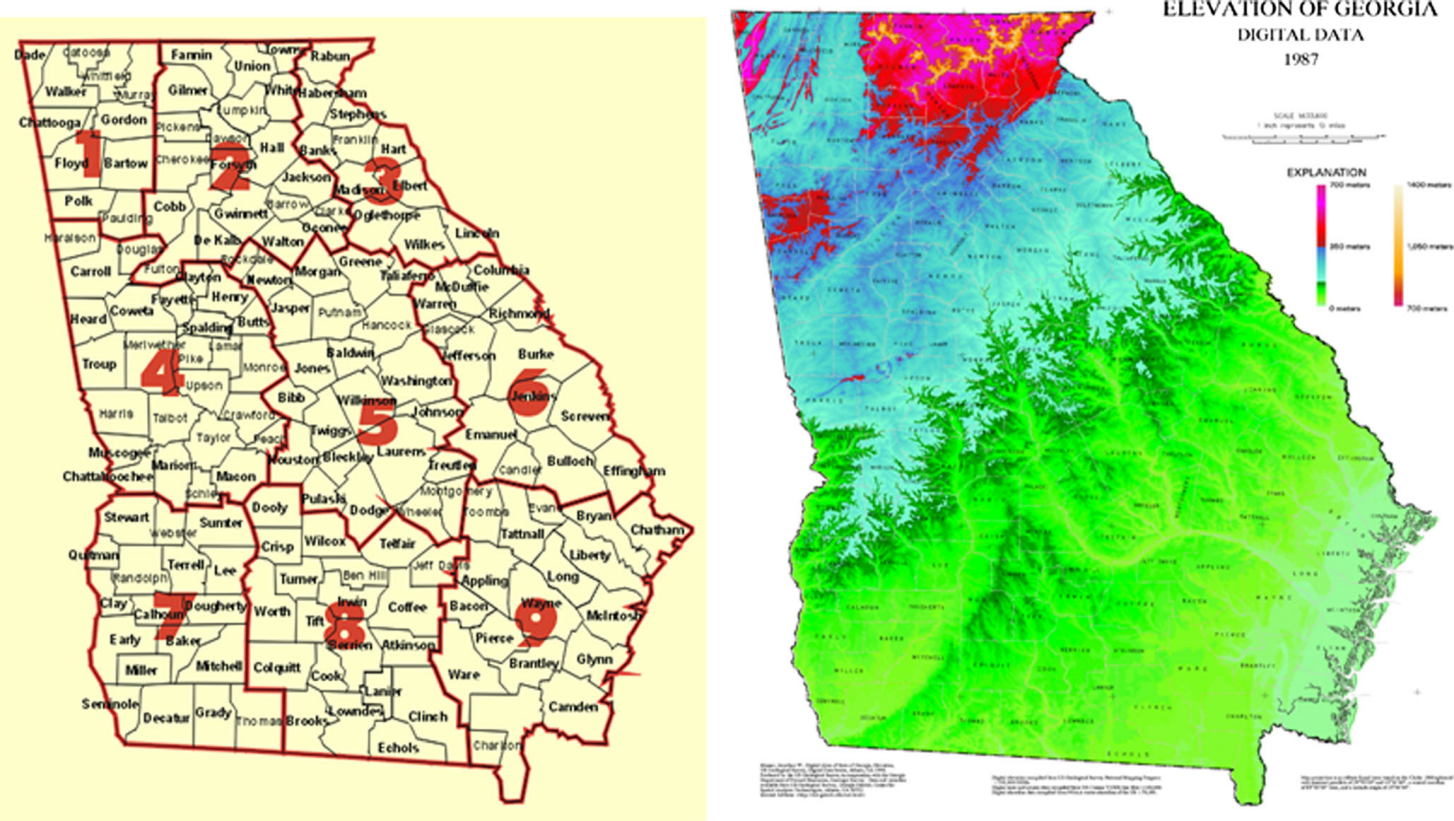
**a** Georgia state map including county (*names*) and climate division (*numerical*) boundaries and **b** digital elevation map highlighting the regions that correspond to mountains (*red*, *pink*), piedmont (*dark blue*), coastal plain (*blue to green* interface), and swamp regions (*yellow to light green*). Maps (public domain access) courtesy of **a** NCDC Climate Prediction Center and **b** U.S. Geological Society

Since the late 1950s the National Climatic Data Center (NCDC) has been recording and assessing temperature, precipitation, and other climate indices based on geographic divisions across the United States (NCDC 2012). Georgia contains nine adjacent divisions (Fig. 1a, courtesy of the NCDC Climate Prediction Center). Division 3 had the lowest population (219,400) and division 2, which includes Atlanta, the largest metropolitan area in the state, had the highest (3,875,000). The average population for the state of Georgia during the study years was approximately 8,828,600.

### Data sources and preparation

Campylobacteriosis case data for the state of Georgia were obtained from the Foodborne Diseases Active Surveillance Network (FoodNet), a program implemented by the Centers for Disease Control and Prevention in Atlanta, Georgia (CDC 2010). A case was defined as a laboratory-confirmed stool or blood sample that tested positive for *Campylobacter*. Names of patients were deleted prior to release of data to investigators and cases identified as travel-related were removed from the dataset to ensure consistent divisional risk estimates. The study was approved by The University of Tennessee Institutional Review Board.

Campylobacteriosis case data were aggregated using SAS version 9.2 into counts, by month per county, over the 10-year study period (1999–2008), resulting in 120 equally spaced data points (SAS Institute 2008). *Campylobacter* case counts were then aggregated at the division level (159 counties share exact boundaries with nine divisions), and merged with annual NCDC division level climate data.

The measure of disease frequency used as the time series variable in the univariate analyses was monthly campylobacteriosis risk. Risk was defined as the probability that an individual will develop campylobacteriosis during the study period (Dahoo et al. 2003). Divisional risks were created in SAS by combining county level US Census Bureau annual population estimates as denominator data (U.S. Census 2010). The risk estimates were presented as the number of cases per 100,000 people, and a very small number (0.01) was uniformly added to the risk to account for a few zero values in the series.

### Pattern analysis, cross-correlations, and outlier identification

The divisional time series were plotted to visualize trend, seasonal patterns, and potential outliers. Autocorrelation (ACF) and partial autocorrelation plots (PACF) were analyzed for seasonal, autoregressive, and moving average patterns. Cross-correlations were calculated between campylobacteriosis risk and precipitation and temperature for each time series. Cross-correlations are a measure of similarity between *X* and *Y* as a function of a time lag (*t* = month) applied to *Y* (*Y*_*t*+*k*_, up to the *k*th order). Kruskal–Wallis one-way non-parametric analysis of variance was used to verify seasonality (*P* < 0.05) in a univariate way, and to identify potential outliers based on monthly medians. Control charts were also used to locate individual outliers (Alwan 1988). Case information on potential outliers was checked for outbreak status. While there is always a potential for outliers to be outbreak-related, outbreak reporting for campylobacteriosis surveillance data may not have been consistently collected throughout the study period.

### Time series analysis

**Fig. 2:**
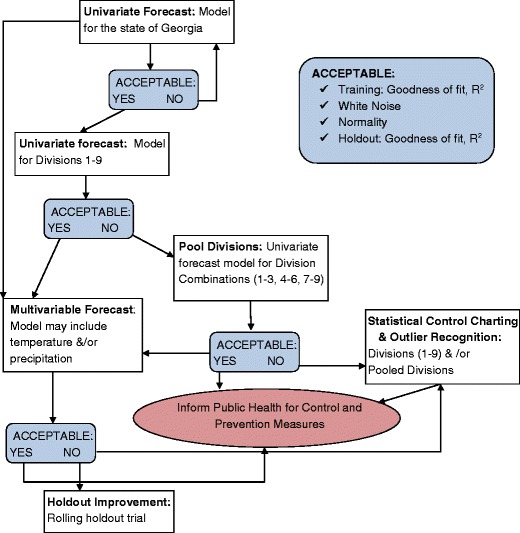
Procedural flow chart for forecasting campylobacteriosis risk in Georgia, USA

A flow chart was used to organize the time series analysis in a logical sequence (Fig. 2). The univariate methods that used monthly campylobacteriosis risk as the only time series predictor were: time series regression, decomposition, Box-Jenkins Autoregressive Integrated Moving Averages (ARIMA), and Winter’s Exponential Smoothing. We also tested temperature and precipitation as univariate predictors of campylobacteriosis risk. The Number Cruncher Statistical System (NCSS) (Hintze 2006) was used for all initial univariate analyses. Models were evaluated for predictive ability and goodness of fit using standardized pseudo *R* ^2^ calculated as one minus the sum of the residuals squared divided by the total sum of squares:

$$ {R^2}_{\mathrm{pseudo}}=1.0-\frac{{\displaystyle \sum_{i=1}^n{\left({y}_i-{\widehat{y}}_i\right)}^2}}{{\displaystyle \sum_{i=1}^n{\left({y}_i-\overline{y}\right)}^2}} $$

While *R* ^2^ is typically associated with ordinary least squares regression, the pseudo *R* ^2^ is a standardized way to compute and compare model fit for any time series technique.

An automated multiplicative model decomposed the series into trend (*T*_*t*_), season (*S*_*t*_), cyclic (*C*_*t*_), and error (*E*_*t*_) components in NCSS as follows:

$$ {Y}_t={T}_t\cdot {S}_t\cdot {C}_t\cdot {E}_t $$

Ordinary least squares regression was used to evaluate the series parameters of trend, month, and year for each division. The best fitting additive time series regression model included the intercept, linear trend, month (seasonal component), and error terms shown respectively below:

$$ {Y}_t={\beta}_0+{\beta}_1{x}_{\mathrm{trend}}+{\displaystyle \sum_{i=2}^{p=12}{\beta}_i{d}_i}+{\varepsilon}_t $$

Multivariable models that included external variables (precipitation and temperature) were compared using time series regression in the SAS forecasting system. Two- and 3-month moving averages for precipitation were investigated for the effect of accumulated rainfall on campylobacteriosis risk. For example, ARIMA patterns or high end outliers (statistically termed interventions) were checked for significance in the model residual. For regression models, variables were retained based on overall model significance, significance of regression coefficients (*P* < 0.05), improvement of predictive value (*R* ^2^), and lack of collinearity.

Division combination datasets were created by averaging division case counts, population data, and climatic data to compare and improve upon forecasting results. The combinations included divisions 1–3, 4–6, and 7–9, and were based on proximity and topographical features (Appalachian mountain range, piedmont and lowland plains, coast, and swamp; Fig. 1b, public domain, courtesy of the U.S. Geological Society) (Netstate 2012; Wikipedia 2012). Modeling and control charting were applied to these series as previously described.

Years spanning 1999 to 2007 were used to model each series. The year 2008 was held out of the data set for model validation as the most recent time series information is typically the most important for prediction purposes (Bergmeir and Benitez 2012). Residual time series plots were checked for normality using the Shapiro–Wilk’s goodness of fit test. The Portmanteau test was used to assess if patterns in the data had been fully extracted during the modeling process and resulted in “white noise”, or random scatter, in the residuals (DeLurgio 1997).

### Statistical control charting and holdout sample variation

Conservative ARIMA (0,0,0)(0,1,1) models were fit for all time series, as process control does not require precise, individual model specification (Alwan 1988). For these data, non-seasonal parameters were not significant. The raw residuals were divided by the root mean square error and scatterplots of the standardized residuals used to identify points that were greater than three standard deviations from zero, or “out of control” (Alwan 1988).

For divisions with known outliers in or near the holdout, or those with poor holdout performance, a moving holdout period that spanned the last 24 months of data (2007–2008) was constructed. Coefficient of determination (*R* ^2^) values were calculated and averaged for five holdouts with 12-month horizons (holdout months 1–12, 4–15, 7–18, 10–21, and 13–24). The training period was evaluated up to the beginning of each of the five holdout periods and the average compared with the holdout results from 2008.

## Results

### Temporal patterns of campylobacteriosis risk

**Fig. 3:**
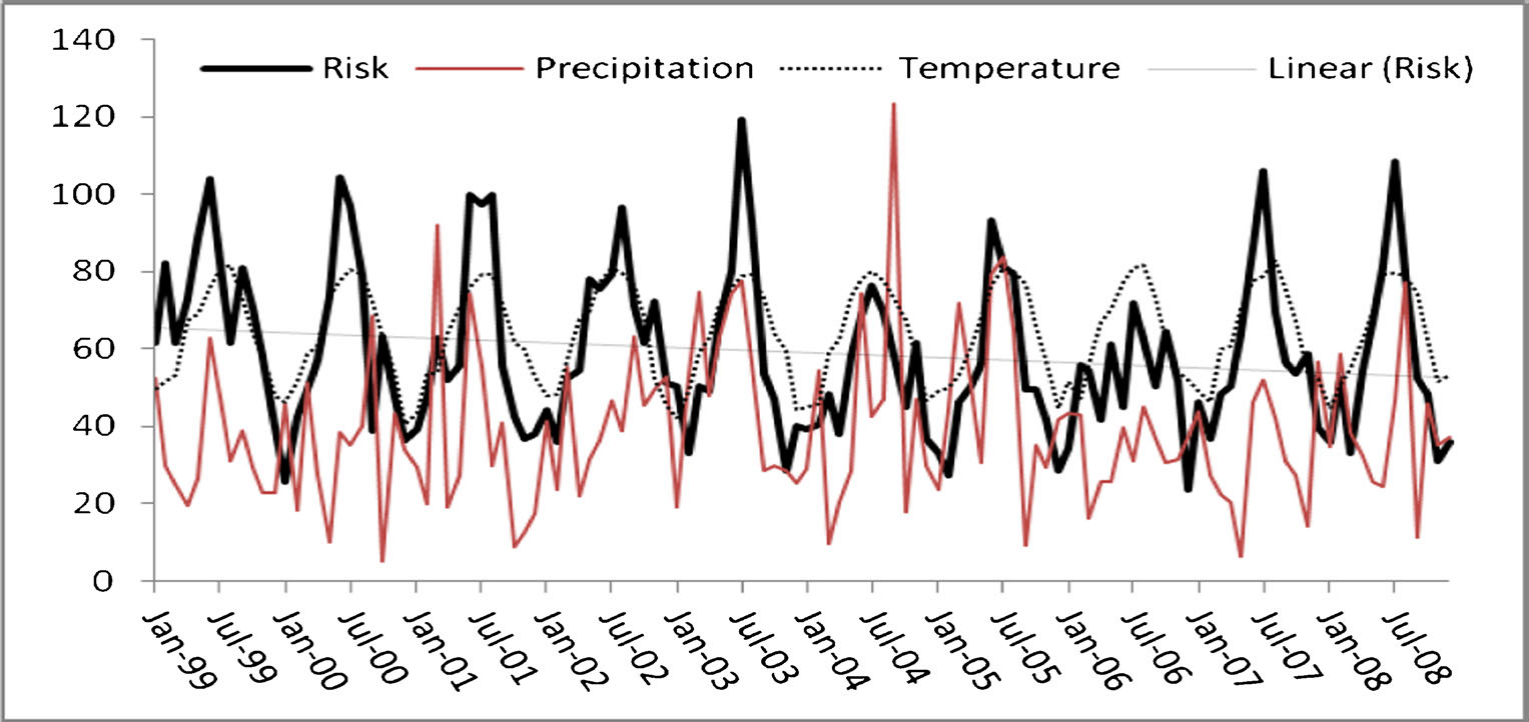
Risk (and linear trend) of campylobacteriosis (scale factor of 100), precipitation (scale factor of 10), and temperature in Georgia, USA from 1999 to 2008

**Fig. 4:**
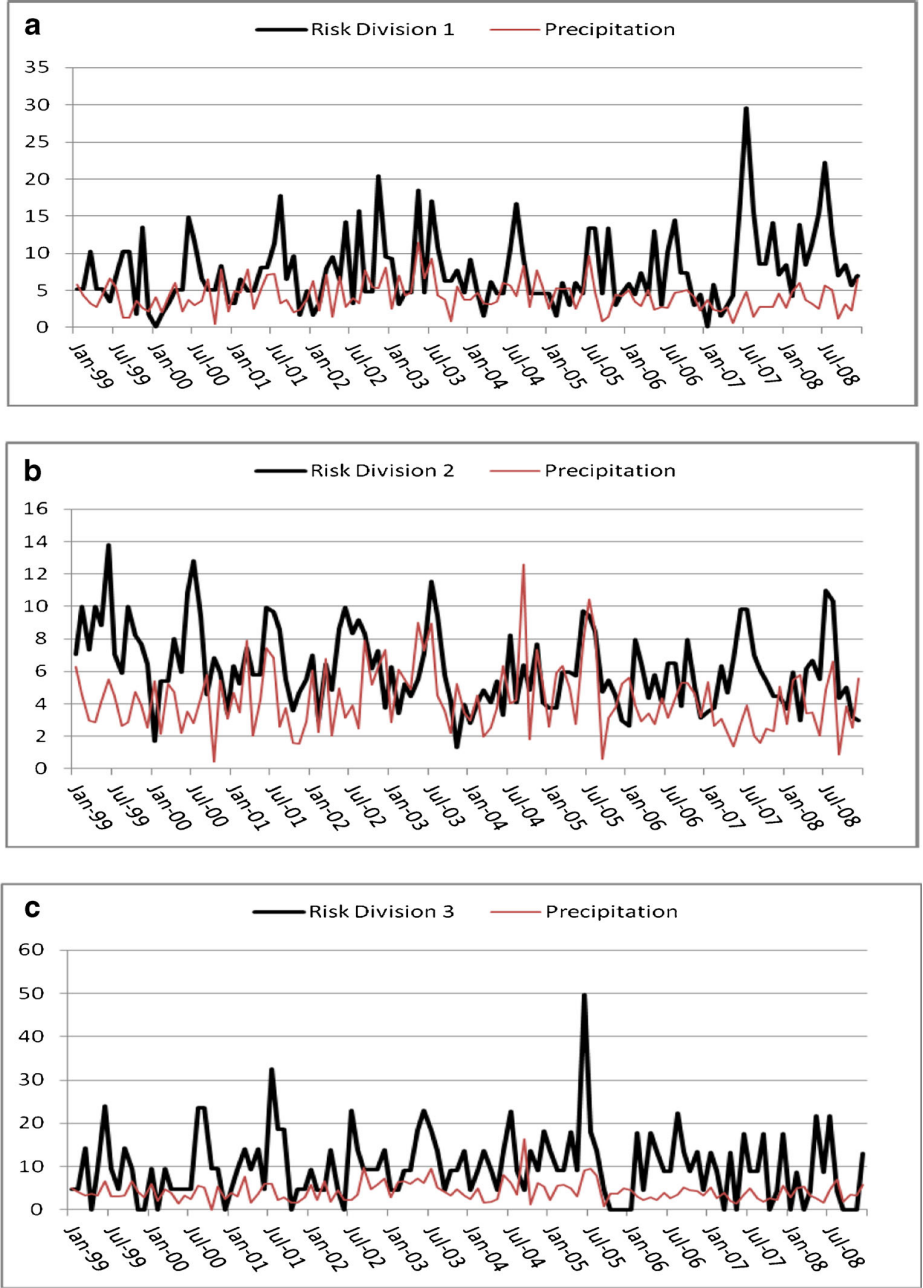

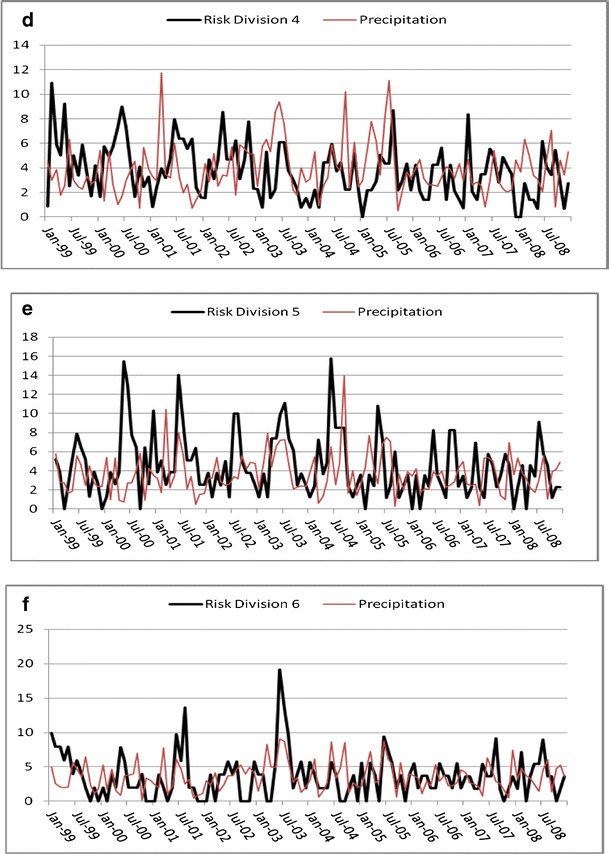

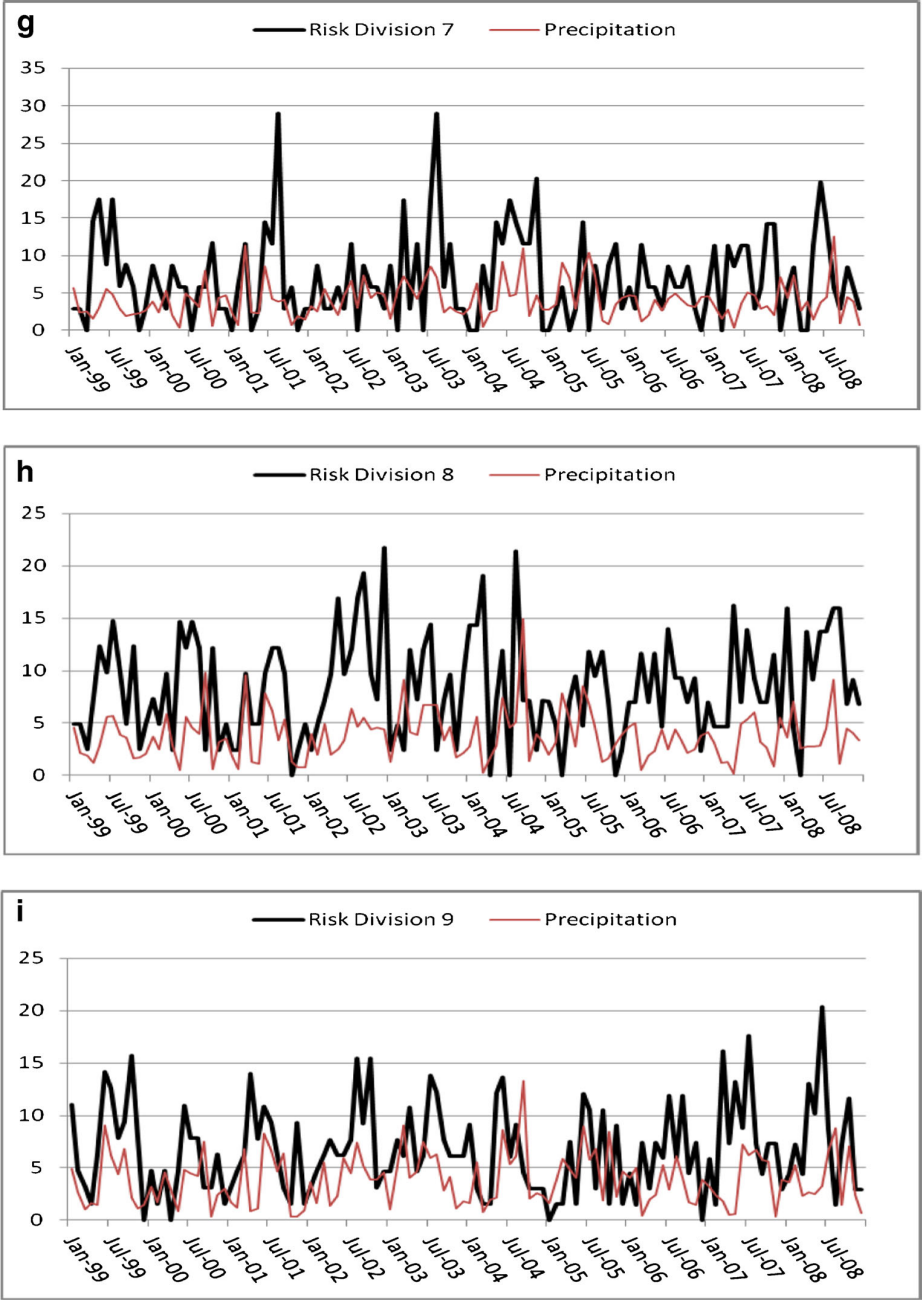
Risk of campylobacteriosis (scale factor of 10) and precipitation for Georgia, USA climate divisions 1–9 (**a**–**i**) from 1999 to 2008

The time series for Georgia campylobacteriosis risk is scaled and displayed with temperature and precipitation and shows a slight downward trend (*P* < 0.01; Fig. 3). Monthly risks ranged from 0.236 to 1.191 per 100,000 (mean = 0.593; Table 1). Division risks were scaled for display with precipitation (Fig. 4a–i), and ranged from 0 (in all divisions except division 2) to 4.97 per 100,000 (division 3; Table 1). Divisions 3 and 5–9 showed no significant trend whereas divisions 2 and 4 had slight downward trends (*P* < 0.01) and division 1 trended slightly upward (*P* < 0.05). Autocorrelation function plots (not shown) indicated a strong seasonal pattern with potential autoregressive and moving average components. The patterns were not clean, implying that there could be outliers, unidentified patterns in the series, or both. Kruskal–Wallis results verified seasonality in all series except division 3, where the median risk between months did not vary significantly (*P* > 0.05).

**Table 1:** Descriptive statistics and cross-correlations (temperature and precipitation) for monthly campylobacteriosis risk estimates per 100,000 population in Georgia, climate divisions 1–9, and combined divisions 1–3, 4–6, and 7–9

Area Division/combination	Risk range per 100,000		Mean risk (standard deviation)	Zero values (# in dataset)	Trend (*P* < 0.05)	Cross-correlations^a^				
						Temperature				Precipitation
						Lag: 0	Lag: 1	Lag: 6	Lag: 12	Lag, CC
Georgia	0.24	1.19	0.59 (0.20)	None	Down	0.71	0.57	−0.72	0.69	0, 0.31
1	0.00	2.97	0.78 (0.50)	2^b^	Up	0.44	0.47	−0.48	0.46	4, −0.15
2	0.13	1.38	0.63 (0.24)	None	Down	0.53	0.41	−0.51	0.52	0, 0.17
3	0.00	4.97	0.99 (0.78)	19^b^	–	0.34	0.26	−0.34	0.32	0, 0.28
4	0.00	1.09	0.37 (0.22)	3^b^	Down	0.40	0.33	−0.44	0.45	8, −0.18
5	0.00	1.58	0.45 (0.33)	8^b^	–	0.43	0.33	−0.50	0.42	0, 0.13
6	0.00	1.92	0.36 (0.32)	25^b^	–	0.31	0.15	−0.39	0.33	0, 0.29
7	0.00	3.90	0.69 (0.59)	18^b^	–	0.35	0.33	−0.31	0.32	0, 0.16
8	0.00	2.18	0.84 (0.49)	6^b^	–	0.38	0.30	−0.35	0.33	1, 0.19
9	0.00	2.03	0.67 (0.43)	4	–	0.50	0.40	−0.45	0.41	6, −0.35
1, 2, and 3	0.22	2.13	0.79 (0.36)	None	–	0.58	0.48	−0.58	0.56	0, 0.31
4, 5, and 6	0.07	1.17	0.40 (0.22)	None	–	0.54	0.38	−0.62	0.54	0, 0.19
7, 8, and 9	0.08	1.58	0.72 (0.34)	None	–	0.60	0.50	−0.55	0.52	0, 0.22

^a^Strongest cross-correlations for temperature (lag 0, 1, 6, and 12) and precipitation (varies) are reported

^b^Some zero values are located in the last 24 months of data (2007–2008)

A consistent seasonal peak between June and August (highest risk in July for most series) was identified in all divisions, including the aggregate state of Georgia. In some divisions, for some years, the peak began early. For example, in the year 2000, risks were higher in May than any other year. The seasonal peak extended into September, sporadically, across divisions and years. Division 3 seasonality did not vary significantly by month, yet the June through August peak is visually evident, although less pronounced, than the other divisions.

To illustrate an example of the unique relationship between campylobacteriosis risk and precipitation, we compared divisions 1 and 9 (Fig. 4a and i) from January 2005 to 2008. We chose these two divisions simply because they represent diverse climatic and geographic regions within the state (Fig. 1a and b). In 2005 when precipitation levels were “normal” (drought categories, in quotes, as determined by U.S. Drought Monitor indices (U.S. Drought Monitor 2012)), risk and precipitation mirror one another in shape and magnitude. In April 2006, Georgia entered a period of “abnormally dry” weather that increased to “moderate drought” by mid-June. By the end of August drought conditions were severe in a majority of the state, and stayed dry, with ranging severity, until the end of 2008. The time series patterns of 2005 may be reflective of normal rainfall. This was followed by risk and precipitation patterns that were muted and divergent, but still cohesive, during the beginning of the drought in 2006. A distinct increase in risk is evident in 2007 that continues throughout 2008 following 2 years of persistent drought conditions. This general pattern is also identified in divisions 2, 7, and 8 (Fig. 4b, g, and h) and at the state level (Fig. 3). During this time period the statewide impact of extreme weather conditions appears to override regional differences in geography and climate. There is no simple visual or statistical way to confirm the exact nature of the relationship between campylobacterioisis risk and precipitation based on these variations in risk. It is possible that the relationship follows a complex cyclic or parabolic curve or is regionally dependent and requires further investigation that is beyond the scope of this study.

### Cross-correlations and outlier identification

Cross-correlations between risk and temperature were strongest at time zero, lags 1, 6, and 12 on all series (Table 1). Cross-correlations for precipitation were weak and highly variable with the highest positive correlation for Georgia at time zero (0.31) and the highest negative correlation in division 9 (−0.35, lag 6). The strong seasonality of campylobacteriosis risk contributed to the strength and timing of the cross-correlations with temperature, and the weak correlations between risk and precipitation may be due to erratic divisional patterns.

Outliers were not identified in the Georgia state level time series. Between one and two outliers were flagged in each division and combination series using Kruskal–Wallis monthly median analysis and/or control charts. All of the divisional series except division 2 contained zero values, notably division 3 (19/120, 15.8 %), division 6 (25/120, 20.8 %), and division 7 (18/120, 15 %; Table 1), yet these low-end values were not statistically significant outliers.

### Forecasting results and comparisons

Time series regression models were significant (*P* < 0.05) globally and for individual model coefficients. The best prediction results were obtained from the aggregated datasets (state of Georgia, then the combinations) followed by divisions 2 and 6 (Table 2). Decomposition and time series regression forecasting results and holdouts were comparable (within *R* ^2^ = 10 %; Table 2), suggesting that one can forecast campylobacteriosis using a classical univariate modeling approach (time series risk data only), or by incorporating external climate variables. In divisions 3, 4, 7, and combinations 1–3 and 4–6, models with external variables, inclusive of outliers showed marginal improvement over decomposition.

**Table 2:** Decomposition and time series regression with external variables and interventions for Georgia, USA, divisions 1–9 and combinations 1–3, 4–6, and 7–9

Division	Univariate regression		Decomposition				Custom time series regression				
	PCP (*P* < 0.05)	TMP (*R* ^2^)	*R* ^2^	Holdout	Norm	White noise	Variables	*R* ^2^	Holdout	Norm	White noise
Georgia	Yes	0.51	0.73	0.66	Yes	No, close	T, PCP, TMP (lag 6)	0.64	0.66	Yes	No
1	No	0.26	0.44	0.13	No, close	Yes	TMP (lag 6) IV: July 2007	0.40	0.02	Yes	Yes
2	Yes	0.30	0.55	0.40	Yes	No	T, PCP, TMP	0.46 Robust^a^	0.48	Yes	Yes
3	Yes	0.12	0.27	–0.37	Yes	No	PCP, TMP IV: June 2005	0.36	0.03	Yes	Yes
4	No	0.17	0.37	0.19	No, close	Yes	T, TMP (lag 6) IV: Jan 2007	0.42	0.26	Yes	Yes
5	No	0.19	0.41	0.09	Yes	Yes	TMP (lag 6)	0.33 Robust	0.31	No, close	Yes
6	Yes	0.10	0.47	0.21	Yes	No	PCP, TMP (lag 6) IV: Aug 2001 IV: June 2003	0.47	0.45	Yes	Yes
7	No	0.13	0.31	0.24	No, close	Yes	TMP IV: Aug 2001 IV: Aug 2003	0.35	0.20	Yes	Yes
8	No	0.15	0.29	0.11	Yes	Yes	TMP IV: Aug 2002	0.27	0.00	No	Yes
9	Yes	0.25	0.36	0.12	Yes	No	PCP (lag 6), TMP	0.31	0.09	No, close	Yes
1, 2, and 3	Yes	0.33	0.49	0.43	Yes	No	PCP, TMP (lag 6) IV: June 2005	0.52	0.46	Yes	Yes
4, 5, and 6	Yes	0.27	0.55	0.43	Yes	No	TMP (lag 6) IV: May 2000 IV: June 2003	0.57	0.34	No, close	Yes
7, 8, and 9	Yes	0.36	0.51	0.22	Yes	No	TMP	0.36	0.28	Yes	Yes

*T* trend, *PCP* precipitation, *TMP* temperature, *IV* intervention

^a^Robust method improved normality and model results

Temperature was significant (*P* < 0.05) in univariate regression models with impact ranging from *R* ^2^ = 10 % in division 6, to 51 % for the state of Georgia (Table 2). Precipitation had a significant positive relationship with campylobacteriosis risk state wide, in the three combinations, and in divisions 2, 3, 6, and 9. The *R* ^2^ contribution of precipitation was less than 10 % when modeled alone and improved model significance when included in multivariable models for Georgia, combinations 1–3, and divisions 2, 3, 6, and 9. The 3-month moving average for precipitation that tested the cumulative effect of rainfall on campylobacteriosis risk decreased the predictive performance (*R* ^2^) of the divisional models by 3–10 %, but improved performance for the state of Georgia by 3 % and combination 4–6 by 5 %.

While the Georgia state model did well on the holdout for both decomposition and time series regression, holdout *R* ^2^ was low for most divisions (*R* ^2^ < 40 %). Decomposition models had more issues attaining white noise than did time series regression (regression models all achieved white noise in the residuals). Most models had residuals with normal distributions (*P* < 0.05). However, in some cases (Table 2), the presence of a few outliers hampered the achievement of normality, which may distort the regression fit and inflate significance tests.

### Statistical control charting and holdout variation results

**Fig. 5:**
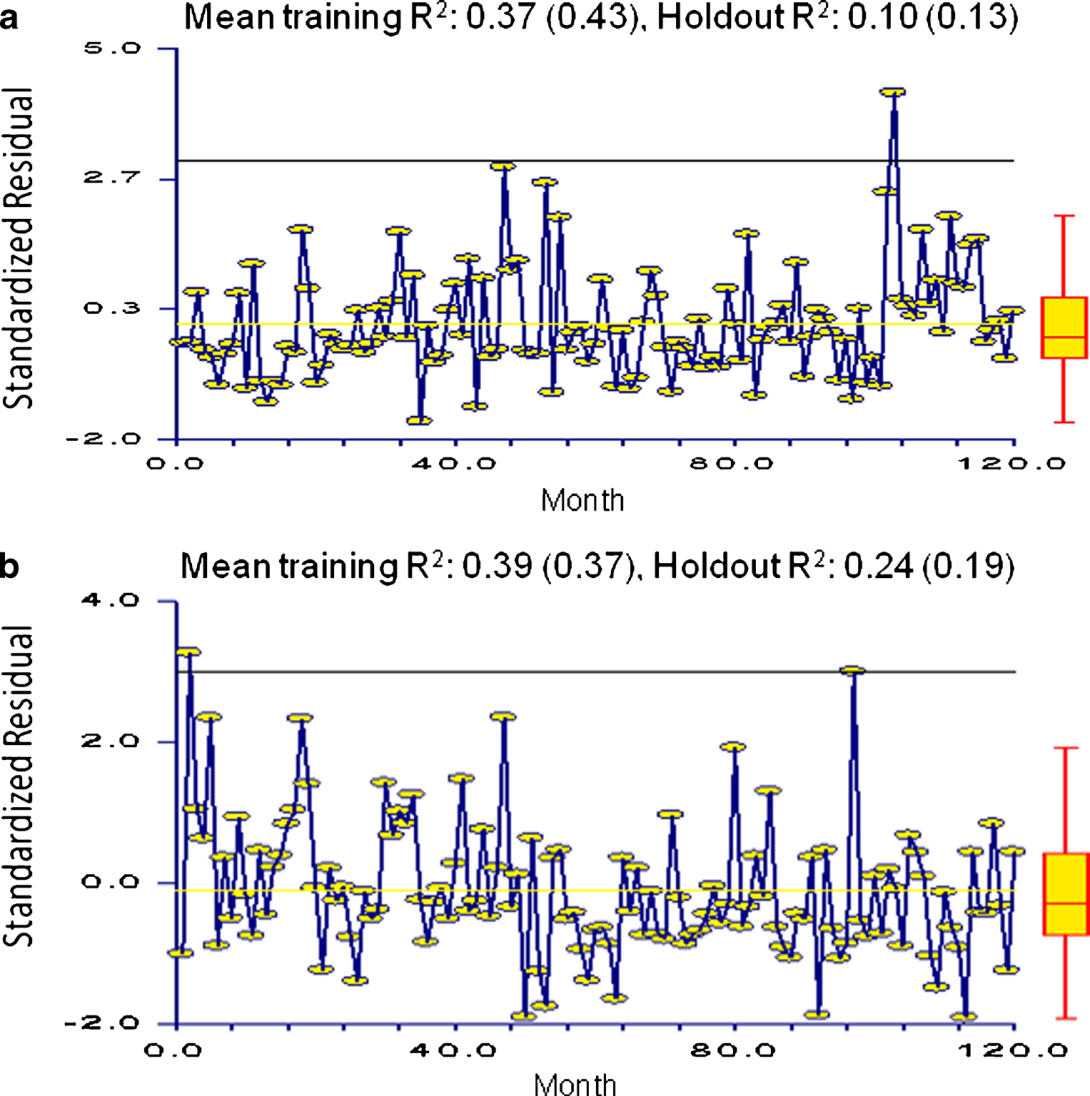
ARIMA (000 011) Control Charting and Moving holdout results for division 1 (**a**) and division 4 (**b**). Included are the standardized residual scatter plots and mean training and holdout *R* ^2^ results for moving holdout analyses using decomposition (compared with 2008 results). **a** Mean training *R* ^2^, 0.37 (0.43); holdout *R* ^2^, 0.10 (0.13). **b** Mean training *R* ^2^, 0.39 (0.37); holdout *R* ^2^, 0.24 (0.19)

Control charting for outlier detection served two important purposes: (1) outliers were able to be incorporated into forecasting models to improve prediction and (2) “out of control” months could be further investigated for division level outbreak potential. Control charts indicated at least one outlier in all divisions except division 9 and combinations 7–9. The flagged observations are as follows: State of Georgia (July 2003), division 1 (July 2007), division 2 (June 1999), division 3 (June 2005), division 4 (February 1999 and January 2007), division 5 (May 2000), division 6 (August 2001 and June 2003), division 7 (August 2001 and August 2003), division 8 (December 2002), combinations 1–3 (June 2005), and combination 4–6 (May 2000 and June 2003). All outliers were high-end (data point above three standard deviations from zero) and most (except those in divisions 4 and 8) occurred during the seasonal spring–summer peak. The moving holdout variation only marginally improved forecasting for division 4, and results for divisions 1 and 9 were worse than those from the initial 2008 holdout sample (Fig. 5). While moving holdout trials proved unsuccessful in improving results, incorporating control charting may be useful for outbreak detection as well as improving forecasting.

## Discussion

The seasonality of campylobacteriosis is well documented worldwide, yet our study is the first to compare univariate and multivariable forecasting approaches in the United States. Our study confirmed a distinct seasonal peak in campylobacteriosis risk between June and August and unique seasonal variation among the nine divisions that was consistent for each over time. Division level variation in risk may result from differences in sources of infection and urban verses rural residential status of individuals (Hearnden et al. 2003; Miller et al. 2004). Weather conditions, temperature, and precipitation may also impact contamination and carriage rates in environmental (animal and water) reservoirs differently from one division to the next. In some studies, environmental sources are responsible for the nearly simultaneous increase in *Campylobacter* infection rates in poultry and human populations (Meldrum et al. 2005; Patrick et al. 2004). The seasonal distribution patterns of *Campylobacter* spp. found in sewage and animal feces, along with seasonal increases in fecal shedding in domestic animals, have also been correlated with human infections (Jones 2001; Stanley and Jones 2003; Stanley et al. 1998; Hutchison et al. 2005). In a Georgia ecological study, the highest *Campylobacter* counts were found in natural waters during the summer (Vereen et al. 2007) coinciding with the highest disease risk found in our study. Isolation from water is indicative of recent contamination, as replication does not occur outside the mammalian host (Eyles et al. 2003). However, it is unclear whether animals and the environment are being seasonally contaminated, re-infected, or subject to natural, biological, or management-related fluctuations in *Campylobacter* over time. To understand transmission routes and disease reservoirs for campylobacteriosis in Georgia it may be necessary to hone in on the environmental, seasonal, and human population characteristics of specific divisions or distinct geographic locations.

Multivariable time series regression models that included climatic factors improved model specification over univariate models—they attained white noise in the residuals. We expected climatic factors to be important. Previous studies have shown these variables to influence disease risk, as well as affect the survival and reproduction of *Campylobacter* spp. in the environment and on food sources (Patrick et al. 2004; Fleury et al. 2006; Tam et al. 2006; Bi et al. 2008). For example, seasonal increases in sunlight hours and warmer temperatures have a direct effect on human activities that increase exposure to *Campylobacter* sources (Hearnden et al. 2003). Known risk factors such as consumption of untreated water, contact with farm animals (Tam et al. 2006; Meldrum et al. 2005), swimming, camping, barbecuing, and other outdoor recreation (Obiri-Danso and Jones 1999; Schönberg-Norio et al. 2004) have been linked with increased seasonal risk and may also be due to changes in environmental sources of contamination (Hearnden et al. 2003). To fully determine how temperature, precipitation, and overall seasonality impact campylobacteriosis risk, it may be necessary to define and investigate an area of study based on the inherent risk factors for disease.

### Temperature

In univariate models, 10 to 50 % of campylobacteriosis risk was attributable solely to temperature (at time zero). These results are consistent with previous research that links warmer temperatures with increases in campylobacteriosis risk (Kovats et al. 2005; Fleury et al. 2006; Allard et al. 2010). Tam and colleagues found that a 1 °C rise in temperature corresponded with a 5 % increase in reported cases, up to 14 °C in England (Tam et al. 2006). In Sweden, mean annual temperature was associated with a slightly increased risk of campylobacteriosis (RR 1.05 [95 % CI, 1.03–1.07]) (Nygard et al. 2004) and in Denmark high temperatures 4 weeks prior to infection were good predictors (*R* ^2^ = 68 %) of human incidence (Patrick et al. 2004). Our models quantified the importance of temperature for the state of Georgia, and demonstrated variation between divisions. Correlations observed between temperature and disease risk may be associated with the presence of migratory birds, insects, and flies that play an important temperature-dependent role in transmission of disease (Guerin et al. 2008; Ekdahl et al. 2005). Temperature has a strong influence on carriage and contamination rates in wild birds, insects, and rodents, as well as survival of *Campylobacter* in the environment (Patrick et al. 2004; Conlan et al. 2007; Sinton et al. 2007). These factors may differ from one division to the next. Further investigation is warranted to determine if the division level seasonal patterns identified in our study provide useful insight into transmission routes, as well as pathogen and host-specific interactions (Lal et al. 2012).

The timing and the extent to which temperature affects campylobacteriosis risk varies considerably worldwide. In a study that included Europe, Canada, Australia, and New Zealand, only temperature increases 10–14 weeks prior to elevations in human infection were statistically significant (*P* = 0.05) (Kovats et al. 2005). In an Australian study, temperatures in subtropical Brisbane were positively correlated with campylobacteriosis whereas in Adelaide, a temperate city, temperature had an inverse correlation (Bi et al. 2008). In our study, seasonality was a likely contributor to the significant correlations in temperature found at lag 0, 6, and 12, as well as the diminishing, yet still significant correlation at lag one. In the multivariable models, temperature at time zero, or at the 6-month lag, outperformed the 1-month lag, suggesting that complex interactions between sunlight, temperature, and humidity combine during these times to enhance division level differences in the effect of temperature on risk. (Patrick et al. 2004). We suspect that the importance of timing and temperature on campylobacteriosis risk may be dependent on the presence of unique regional or local disease reservoirs, be they natural (i.e., water, wild animals) or anthropogenic (i.e., agriculture, land use, runoff). Local public health officials must become knowledgeable about respective regional environments in order to identify and investigate important variations in risk factors for campylobacteriosis.

### Precipitation

Precipitation patterns for the divisions correspond visually with campylobacteriosis risk during isolated sets of months, despite wide, erratic variations over the 10-year study period (Fig. 4a–i). This suggests that precipitation may be an important baseline factor and that divergent patterns are due to unknown local factors and spikes in campylobacteriosis risk. The predictive ability of precipitation (<10 % *R* ^2^) was similar to that of a Denmark study in 2004 where precipitation explained 6 % of the variation in human incidence (Patrick et al. 2004). As indicated by our case study of years 2005–2009 (divisions 1 and 9), climatic factors may combine to invoke a “threshold” at which the cumulative impact of summer induces an exponential biological or behavioral increase in the risk of campylobacteriosis, or causes outbreaks that are undetected by current surveillance.

In regions where summer is characterized by increased rainfall and flooding, pathogen levels are magnified as fecal material is transferred from land to surface waters (Eyles et al. 2003; Coker et al. 2001). Our data showed an increase in risk during drought. While rainfall may improve survival and leaching of the bacteria, drought conditions concentrate bacterial loads (Patrick et al. 2004; Sinton et al. 2007). Northeast Georgia is well known for livestock production, specifically intensive poultry farms. Local investigation of the environmental impact of the animal production industry and its effects on human populations at risk may lead to a better understanding of the relationship between precipitation and campylobacteriosis in this area.

Results of the 3-month moving average for precipitation (improved predictions for the state of Georgia and worsened divisional predictions) suggest that the predictive sense may be unique to each division. For example, smoothing techniques diluted the association between campylobacteriosis risk and precipitation at the division level. Significant division level relationships between precipitation and campylobacteriosis risk in our study may explain why studies that use data over large geographic areas observe no such association (Kovats et al. 2005). Analogous to a magnifying lens, whereby “zooming out” (smoothing or aggregating data), conveys a broader sense of the relationship, “zooming in” captures the detail, as well as the anomalies. Public health officials should objectively and qualitatively assess time periods where campylobacteriosis risk diverges from typical precipitation patterns and investigate the potential causes. In doing so, decisions for control and prevention are based on empirical evidence.

### Statistical control charts and outliers

In our study, control charts served to focus attention on specific time periods of high campylobacteriosis risk. While it may be useful to identify erratic points in an aggregated dataset, the division level control charts narrow the focus of investigation. For example, May 2000 and June 2003 were flagged in the combination 4–6 series, and further identified in divisions 5 and 6, respectively. Traditional models often incorporate smoothing, robust methods or model outliers to meet assumptions, thereby sculpting a state of statistical control. According to Alwan and Roberts, improving our ability to distinguish between special causes and common causes (in this case, for example, outbreaks vs. known seasonality) justifies the effort to identify an “out of control process” (Alwan 1988). Alternatively, statistical control charting can be used as a guideline to assess expected vs. observed level of risk (Benneyan 2001) and serve to augment predictive models when patterns are erratic.

In the poorest performing divisions with outliers (divisions 1 and 4) and without outliers (division 9) we were unable to identify the point of tension in the series whereby the analysis could be improved or refined with a moving holdout. Excessive variation, randomness or outliers in the 24-month holdout period, holdout or horizon lengths, or a combination of these may have contributed to the lack of improvement. While outliers near the end of a series or in a holdout can have severe effects on forecasting ability and accuracy (Bergmeir and Benitez 2012), incorporating moving holdouts may be impractical in the realm of public health when model improvement is not a guarantee, and the process requires expertise and can be time consuming. We recommend that these methods be reserved for time series with forecasts that are significantly improved (>10 % *R* ^2^) as a result of accounting for such outliers.

### Limitations

Considerable variation was observed in results among divisions and irregular patterns, randomness, or outliers decreased forecasting accuracy (Makridakis and Hibon 1979). The presence of outliers or change points can alter patterns and invalidate forecasts and the effect of zero values in these series is unclear. Low or high disease counts and potential outbreaks should be verified and addressed using multiple techniques and sources of information when available. In addition, the use of external variables for forecasting increases model uncertainty. In these situations, a decision must be made as to what objectives are most important—accurate predictions or understanding biological and epidemiological patterns.

The divisional datasets illustrate potential real-life dilemmas such as small number problems, unidentified outbreaks, variations, and inconsistencies inherent in surveillance data. *Campylobacter* infection is typically self-limiting and people who become ill do not always seek care. In general, surveillance systems tend to underestimate actual disease risk and reporting may vary between states and divisions, therefore predictions should be interpreted with caution. However, these data were actively ascertained and thought to represent the burden of culture-confirmed campylobacteriosis (Scallan et al. 2011).

The models and divisions varied in the ability to achieve normality and/or white noise in the residuals. Lack of white noise may increase model uncertainty or indicate misspecification, as patterns in the residuals are not accounted for. However, models without perfect attainment of white noise can have valid results (Burkom et al. 2007), and poor predictive performance was likely due to outliers as opposed to issues of model specification.

## Conclusions

Division level climatic factors and statistical control charting improve models for predicting monthly campylobacteriosis risk, particularly when there are erratic, non-random patterns in the time series. Control charts can serve as an early warning system by detecting irregularities in campylobacteriosis risk at the division level. Forecasting on aggregated data (the state of Georgia, or combinations of divisions) improved our explanation of the variance in risk in decomposition models and multivariable time series regression. However, division level patterns and their associations with temperature and precipitation highlight how important information can be gleaned from finer levels of analysis. Public health officials can more readily address unusual high risk estimates in a timely manner by focusing investigations on the causal factors that relate directly to the region of interest. Climate information can be rapidly and freely obtained, therefore assessing the baseline impact of temperature and precipitation, may be a useful “real-time” alternative for those who do not have ready access to surveillance data. The descriptive information, temporally housed in empirical *Campylobacter* disease risk data may also improve our understanding of biological, environmental, and behavioral drivers of disease. Furthermore, modeling and comparing campylobacteriosis patterns among divisions with different environmental characteristics may lead to the identification of unique disease transmission routes, sources, and reservoirs.
